# Epstein-Barr virus-specific methylation of human genes in gastric cancer cells

**DOI:** 10.1186/1750-9378-5-27

**Published:** 2010-12-31

**Authors:** Julie L Ryan, Richard J Jones, Shannon C Kenney, Ashley G Rivenbark, Weihua Tang, Elizabeth RW Knight, William B Coleman, Margaret L Gulley

**Affiliations:** 1 Departments of Dermatology & Radiation Oncology, University of Rochester Medical Center, Rochester, NY 14642-8704, USA; 2 Department of Lymphoma and Myeloma, MD Anderson Cancer Center, Houston, TX 77054, USA; 3 Departments of Oncology and Medicine, McArdle Laboratory, University of Wisconsin School of Medicine and Public Health, Madison, WI 53706, USA; 4 Department of Pathology and Laboratory Medicine and the Lineberger Comprehensive Cancer Center, University of North Carolina, Chapel Hill, NC 27599-7525, USA

## Abstract

**Background:**

Epstein-Barr Virus (EBV) is found in 10% of all gastric adenocarcinomas but its role in tumor development and maintenance remains unclear. The objective of this study was to examine EBV-mediated dysregulation of cellular factors implicated in gastric carcinogenesis.

**Methods:**

Gene expression patterns were examined in EBV-negative and EBV-positive AGS gastric epithelial cells using a low density microarray, reverse transcription PCR, histochemical stains, and methylation-specific DNA sequencing. Expression of PTGS2 (COX2) was measured in AGS cells and in primary gastric adenocarcinoma tissues.

**Results:**

In array studies, nearly half of the 96 human genes tested, representing 15 different cancer-related signal transduction pathways, were dysregulated after EBV infection. Reverse transcription PCR confirmed significant impact on factors having diverse functions such as cell cycle regulation (*IGFBP3*, *CDKN2A, CCND1, HSP70, ID2, ID4)*, DNA repair *(BRCA1, TFF1*), cell adhesion (*ICAM1*), inflammation (*COX2*), and angiogenesis (*HIF1A*). Demethylation using 5-aza-2'-deoxycytidine reversed the EBV-mediated dysregulation for all 11 genes listed here. For some promoter sequences, CpG island methylation and demethylation occurred in an EBV-specific pattern as shown by bisulfite DNA sequencing. Immunohistochemistry was less sensitive than was western blot for detecting downregulation of COX2 upon EBV infection. Virus-related dysregulation of COX2 levels *in vitro *was not recapitulated *in vivo *among naturally infected gastric cancer tissues.

**Conclusions:**

EBV alters human gene expression in ways that could contribute to the unique pathobiology of virus-associated cancer. Furthermore, the frequency and reversability of methylation-related transcriptional alterations suggest that demethylating agents have therapeutic potential for managing EBV-related carcinoma.

## Background

Gastric cancer is the fourth most common type of cancer and the second leading cause of cancer death worldwide [1]. A variety of genetic alterations as well as infectious and other environmental agents appear to be factors in gastric carcinogenesis. Epstein-Barr virus (EBV), a double-stranded DNA gammaherpesvirus, is found within the malignant cells in 10% of gastric adenocarcinomas, and infection seems to precede malignant transformation [2]. Basic and clinical observations suggest that EBV-associated gastric cancers have a different pathobiology from EBV-negative gastric cancers [3-8]. Rational design of virus-directed therapy requires a better understanding of the pathogenic role of EBV in gastric carcinogenesis.

Prior studies have shown loss of three critical tumor suppressor gene products, CDH1 (E-cadherin), p73, and CDKN2A (p16), in EBV-infected gastric cancers [9-18]. Virus-associated methylation of these genes, along with evidence of global DNA methylation in EBV-positive cancers, suggests that EBV-related gastric cancers are a subset of CpG island methylator phenotype (CIMP) cancers [4,11,19-26]. A potential mediator is DNA methyltransferase 1 (*DNMT1) *that is upregulated in naturally infected gastric cancers and could help establish methylation patterns propagated to daughter cells upon cell division [21,27-29]. Ongoing studies are aimed at understanding the role of EBV and Helicobacter pylori infection in causing inflammation and associated global hypermethylation during gastric cancer development [22].

In cell line models, DNMT1 overexpression is mediated by EBV LMP1 and LMP2 [21,28-31]. EBV seems to employ epigenetic mechanisms to control the host transcriptome and also to control expression of its own virally encoded genes [11,12,14,15,19,21,24,29,32,33]. Upon initial infection of a cell, the unmethylated viral genome can undergo viral replication with new virion production, while a subset of infected cells acquire a highly methylated viral genome that squelches expression of foreign proteins and mediates long-term viral persistence by way of latent infection [23,34]. Infected tumors tend to have highly methylated EBV DNA, and methylation-related silencing of viral genes helps explain how infected tumors evade immune destruction.

While methylation of gene promoters is typically associated with transcriptional *downregulation *via selective binding of repressor proteins, the first protein ever shown to bind and *activate *a methylated promoter was EBV BZLF1, the key factor controlling the switch from latent to replicative forms of viral infection [35]. It appears that the virus has cleverly evolved a means of overcoming promoter methylation to its advantage [34,35]. Antiviral strategies are being explored for their antineoplastic potential. Interestingly, the most commonly used antiviral agents, acyclovir and ganciclovir, are effective at shutting down viral replication but they do not eliminate expression of latent and early lytic viral genes such as LMP1, LMP2 and BZLF1.

The clinical implications of EBV-related methylation of the gastric cancer genome are immense. First, emerging evidence shows the potential for improved diagnosis of gastric cancer by testing gastric washes for cancer-specific methylation patterns, perhaps in concert with tests for EBV to identify the virus-infected subset of cancers [36-40]. Differing patterns of promoter methylation in virus-positive compared to virus-negative cells [11,21,24] emphasize the need to characterize methylation patterns in a manner that that maximizes assay sensitivity for cancer detection. Both infection and altered DNA methylation appear to be early events in carcinogenesis [2,41], potentially facilitating detection of precancerous lesions in stomach juice.

A second clinical implication is the potential for improved treatment of gastric cancer using drugs that reverse the effect of promoter hypermethylation [42,43]. In particular, demethylating agents that inhibit DNA methyltransferase and reverse tumor suppressor gene silencing or oncogene activation are potential antineoplastic strategies [43]. Consideration must be given to possible differences in the effect of demethylating agents in virus-positive *versus *virus-negative tumors [43-45]. We and others have shown that naturally infected gastric cancers have lower CDKN2A (p16) expression [14,15]. In a clinical trial of fluorouracil (5FU) for gastric cancer, *CDKN2A *promoter methylation status was an independent predictor of survival [46]. The rationale for using demethylating agents like 5-aza-2'-deoxycytidine in clinical trials rests on scientific evidence that demethylating therapy modifies the tumorigenic properties of cancer cells.

Several investigators have successfully infected epithelial cell lines with EBV *in vitro *[47,48]. In the current study, EBV-positive and EBV-negative AGS gastric cancer cells were examined for differences in gene expression patterns using low-density microarray analysis and reverse transcription polymerase chain reaction (rtPCR). AGS is a cell line that was originally grown from gastric adenocarcinoma tissue and now is widely used as a model of gastric cancer. The role of DNA methylation in mediating selected effects was examined by bisulfite DNA sequencing and by testing the ability of a demethylating agent to reverse the effect of EBV on gene silencing. Results revealed extensive gene dysregulation upon EBV infection in AGS cells with evidence that promoter methylation is responsible, at least in part. Reversal of virus-associated transcriptional effects suggests that demethylating agents should be explored for their potential to control growth of EBV-related malignancies.

## Methods

### Gastric Cancer Cell Lines

The AGS gastric cancer cell line (ATCC CRL-1739) was cultured in Dulbecco's Modified Eagle's Medium containing 10% fetal bovine serum (heat-inactivated for 20 minutes at 65°C) and 1% penicillin-streptomycin (10,000 units penicillin, 10,000 μg/ml streptomycin, Gibco, Carlsbad, CA). The cells were infected with a recombinant EBV strain (a gift from Dr. Henri J. Delecluse)[33,49,50] that was engineered to express green fluorescence protein (GFP) and hygromycin B resistance by cloning these genes into the prototypic B95.8 strain of EBV where the second copy of oriLyt normally resides. Before infection, AGS cells were transfected with 1 μg of an expression vector encoding CD21 (the EBV receptor) and a puromycin resistance gene by using Fugene 6 (Roche, Indianapolis, IN) as previously described [51]. At 48 hours post-transfection, cells containing CD21 were positively selected for using 0.5 μg/mL of puromycin-HCl (Roche). Viral stocks of recombinant EBV were generated in kidney 293 cells, a human embryonic epithelial cell line, by inducing lytic replication using 20 ng/mL phorbol 12-tetradecanoate 13-acetate and 3 mM butyrate. Supernatants were harvested 3 days after induction, filtered (0.8 μM), and frozen at -80°C until use. Puromycin-resistant AGS cells were plated at 50% of full density in 60-mm tissue culture dishes and co-incubated with 1 mL of stock virions. Four days later, the EBV-infected AGS cells (now called AGS-B95-HygB) were positively selected with 100 μg/mL hygromycin B (Roche).

DNA fingerprinting confirmed that AGS cells used in this study matched the genotype of AGS cells in the American Type Culture Collection. Fingerprinting was done using the PowerPlex 1.2 STR kit (Promega) followed by electrophoresis on an ABI 310 capillary gel electrophoresis instrument (Applied Biosystems).

### Gene Expression by Histochemical Stains

Paraffin blocks were prepared from AGS and AGS-B95-HygB cell lines, and paraffin blocks of primary gastric adenocarcinoma were retrieved from clinical archives. The residual clinical specimens represent all available EBV positive gastric cancers (n = 9) and a random selection of EBV-negative gastric cancers (n = 9). Histochemical stains were applied to paraffin sections to confirm infection and to evaluate gene expression. To prepare blocks, cultured cells were first rinsed in Dulbecco's phosphate buffered saline (Gibco, Invitrogen), harvested with 0.25% trypsin (Gibco, Invitrogen), enmeshed in a clot using Dade Ci-Trol Coagulation Control (Citrated)-Level 1 (Dade Behring, Marburg, Germany) and Thrombin 200 (Pacific Hemostasis, Middletown, VA), fixed in 10% buffered formalin, embedded in paraffin, and sectioned onto coated slides.

*EBER in situ *hybridization was performed using fluorescein-labeled *EBER *probe and oligo(d)T control probe on a Benchmark *in situ *hybridization system (Ventana Medical Systems, Tucson, AZ). Immunohistochemical stains for EBV LMP1 and LMP2 proteins were performed as previously described [52] using citrate antigen retrieval and the CS1-4 cocktail of mouse monoclonal antibodies against LMP1 (1:100, Dako, Capinteria, CA) and the E411 rat monoclonal antibody against LMP2 (1 mg/ml, Asencion, Munich, Germany). Paraffin sections of EBV-related Hodgkin lymphoma and post-transplant lymphoproliferative disorder served as positive controls.

Immunohistochemical analysis of the EBV replicative proteins BMRF1 and BZLF1 was performed using anti-BMRF1 clone G3-E31 (1:200 dilution, Research Diagnostics, Inc., Flanders, NJ) and anti-BZLF1 clone BZ.1 (1:25 dilution, Dako, Carpinteria, CA), while human PTGS2 (colloquially named cyclooxygenase-2, COX2) was assayed using anti-COX2 monoclonal antibody (1:200 dilution, Cayman Chemical). Sections were incubated with primary antibody for 30 minutes at 37°C for the EBV targets, or at 4°C overnight for COX2, using the blocking and detection protocols in the Super-Sensitive Non Biotin HRP Detection Kit (Biogenex). Bound antibody was detected using diaminobenzidine chromogen (Biogenex) and cells were counterstained with hematoxylin (Dako). Paraffin sections of oral hairy leukoplakia served as a positive control for lytic EBV, while reactive tonsil and nasopharyngeal carcinoma tissues served as controls for COX2 stains. Results were interpreted by microscopic scoring of malignant cells in ≥10 fields at 400x magnification yielding an average proportion score (0 = none, 1 = <1%, 2 = 1-10%, 3 = 10-33%, 4 = 33-66%, 5 = >66% of cells) and intensity score (0 = none, 1 = weak, 2 = intermediate, 3 = strong staining). Total scores were compared in EBV positive *versus *negative tumors using a Mann-Whitney unpaired t test.

### Detection of the EBV genome

A battery of quantitative real-time PCR (Q-PCR) assays targeting six disparate regions of the EBV genome was used to demonstrate that viral infection of AGS cells was successful. Amplified products were detected using the ABI Prism 7500 Real-Time PCR instrument with Sequence Detection System software (Applied Biosystems) as previously described using primers targeting the *BamH1W*, *EBNA1*, *LMP1*, *LMP2*, and *BZLF1 *regions of the EBV genome [52] or *EBER1 *DNA [53]. To check for amplicon contamination, every run contained at least two "no template" controls in which nuclease-free H2O was substituted for template.

### Low-Density cDNA Microarray Analysis

RNA was isolated from AGS and AGS-B95-HygB cells using the RNeasy RNA Mini Kit (Qiagen) after first using the QiaShredder™ spin column (Qiagen) to lyse the cells. After confirming RNA integrity using an Agilent Bioanalyzer, expression microarray analysis was performed by SABiosciences Corporation (Frederick, MD) using their GEArray Q Series Human Signal Transduction in Cancer Gene Array. This low-density microarray consists of 96 probes that test activation of 15 signal transduction pathways involved in oncogenesis. (Target transcripts are listed in Results.) Biotin-labeled cDNA prepared from 10 μg of each RNA sample using the AmpoLabeling-LPR method (SABiosciences) was hybridized to the array, and chemiluminescent detection was performed using a CCD camera. Integrated raw intensity values for each spot were generated by GEArray Analysis Suite software (SABiosciences), and further analysis and normalization was performed using Microsoft Excel. The lowest spot intensity value on each array was considered to be background and was subtracted from each raw intensity value for each probe, and then spot intensities were normalized to that of the housekeeping gene, glyceraldehydephosphate dehydrogenase (*GAPDH*). A pairwise comparison of gene expression levels was made between the EBV-positive cells (AGS-B95-HygB) and parental EBV-negative AGS cells. If the ratio was ≥2 or ≤0.5, the gene was considered to be upregulated or downregulated in infected cells.

### SYBR Green Semiquantitative rtPCR

To confirm selected microarray gene expression results, rtPCR was performed using Real-Time RT^2 ^gene-specific PCR primers (SABiosciences). The ReactionReady™ First Strand cDNA Synthesis Kit (SABiosciences) was used to convert 3 μg of RNA to cDNA, and the cDNA was diluted 1:10 before PCR analysis. The following 38 cDNAs were targeted: *GAPDH, A2M, ABCB1, BCL2L1, BIRC1, BIRC2, BIRC3, EN1, GADD45, HIF1A, ID2, IGFBP3, BRCA1, TMEPAI, IRF1, BCL2, BMP4, CDKN2A (p16), FN1, HK1, ICAM1, IL2, CCND1, MDM2, COX2 (PTGS2), TFRC, WISP1, TRAF1, CDK2, VCAM1, CDKN2C (p18), CDKN1A (p21), DUSP1, HSP70, NFKB1, TNFSF10, TRIM25*, and *FOSL1*. Reactions were performed in a total volume of 25 μL containing 1X TaqMan^® ^Universal Mix (ABI), RT^2 ^gene-specific primer set mix (10 μM each primer, SABiosciences), 2.5 μL 5X SYBR green solution (Molecular Probes, Eugene, OR), and 5 μL of cDNA template. Thermocycling conditions were: 50°C for 2 minutes, 95°C for 10 minutes, and 40 cycles of 95°C for 15 seconds and 60°C for 1 minute on an ABI 7500 instrument with Sequence Detection System software (Applied Biosystems). The same threshold was used for each gene and plate evaluated by the "Relative Quantification Plate" protocol. Each PCR reaction was run in triplicate for each sample on two separate 96-well plates, and results were averaged to determine relative differences in each gene's expression level in the EBV-positive *versus *EBV-negative cell line.

### Minor Groove Binding Probe Semiquantitative rtPCR

To evaluate expression of five selected genes that were not on the microarray described above, semi-quantitative rtPCR assays were performed (Assays-on Demand, Applied Biosystems) using minor groove binding probes targeting helicase-like transcription factor (*HLTF*), trefoil factor-1 (*TFF1*), basic-leucine zipper ATF-like transcription factor (*BATF*), inhibitor of DNA binding protein-4 (*ID4*), and nucleostemin (*NU*). These five were selected because they are reportedly dysregulated in a substantial proportion of gastric adenocarcinomas or EBV-related cancers [32,54-59]. *GAPDH *served as an endogenous control for relative quantification purposes. RNA was converted to cDNA using the High Capacity cDNA Archive Kit (Applied Biosystems), and the cDNA was diluted 1:10 with nuclease-free water. Each 50 μL PCR reaction contained: 1X TaqMan^® ^Universal Master Mix, 1X Target Gene Expression assay or *GAPDH *Endogenous Control mix, and 10 μL cDNA. To check for amplicon contamination, every expression assay on every plate contained at least two "no template" controls in which nuclease-free water was substituted for template. Thermocycling conditions and data analysis were as described above for the SYBR Green rtPCR.

### Demethylation Treatment and Sequencing of Bisulfite-modified DNA

To study the effect of demethylation, the AGS and AGS-B95-HygB gastric cancer cell lines were grown to 75% confluency and then for three consecutive days 1 μM of fresh 5-aza-2'-deoxycytidine (5aza; Sigma) was added daily. On the fourth day, RNA and DNA were harvested from treated and untreated cultures. RNA was evaluated for gene expression levels, and DNA was examined for methylation after sodium bisulfite treatment (EZ DNA Methylation Kit, Zymo Research, Orange, CA) to convert unmethylated cytosines to uracil, while keeping methylated cytosines unchanged [60]. The positive control was CpGenome Universal Methylated DNA (Chemicon) subjected to the same bisulfite conversion, and control primers targeting the *C8orf4 *cellular gene promoter confirmed successful bisulfite conversion of each DNA sample [61]. CpG islands were identified using CpG Plot for each of five human genes-- *ICAM1 *(RefSeq# NM_00201), *CDKN2A (p16) *(RefSeq# NM_000077.3), *ID4 *(RefSeq# NM_001546), *COX2 *(RefSeq# NM_000963), and *TFF1 *(RefSeq# 003225.2)-- for which promoter sequences (3000 basepairs upstream of the transcription start site through exon 1) were downloaded from GenBank. The following parameters for CpG island identification were used: minimum length of 200 bp, minimum average percentage of C+G of 50%, and minimum average ratio of observed to expected C+G of 0.6 [62]. To identify CpG dense regions for *COX2 *and *TFF1*, the minimum length parameter was reduced to 50 bp [61]. Primer sequences are shown in Table 1. Each 50 μl PCR reaction contained: 1X PCR Buffer, 2 mM MgCl_2_, 1 unit Platinum Taq DNA Polymerase (Invitrogen, Carlsbad CA), 0.2 mM dNTPs (ABI), and 30 pmol of each primer. Thermocycling conditions were: 94°C for 2 minutes, 40 cycles of 94°C for 30 seconds, 55°C for 30 seconds, and 72°C for 1 minute, 72°C for 10 minutes. To monitor amplicon contamination, every run contained a "no template" control in which nuclease-free water was substituted for template

**Table 1:** Bisulfite Converted Gene-Specific PCR Primer Sequences

**ICAM1**	Forward	5'-TGG GGG TTG TGG TTT TAG TT-3'
	Reverse	5'-CTC CCT CCA CTA AAA AC-3'
	Amplicon size	412 bp
**CDKN2A (p16)**	Forward	5'-AGA TGT TTT GTG GTT GTT GTG A-3'
	Reverse	5'-CAA AAA TCT TCC ATT CTT CAA AC-3'
	Amplicon size	418 bp
**ID4**	Forward	5'-TTT TTT GGG TAT ATA TTA GTT TGG-3'
	Reverse	5'-TAT CCT AAT CAC TCC CTT C-3'
	Amplicon size	477 bp
**COX2**	Forward	5'-TAT GTG TTG TAT ATA GAG TAG A-3'
	Reverse	5'-AAA AAA TAA TCC CCA CTC TC -3'
	Amplicon size	399 bp
**TFF1**	Forward	5'-TTA GGT TGG AGT GTA GTA GG-3'
	Reverse	5'-CCT ACT CAT ATC TAA AAA ACC C-3'
	Amplicon size	489 bp
**C8orf4 control**	Forward	5'-GAA TTA AAA TAT AAG GAG AGT TTT-3'
	Reverse	5'-AAC ATT ACC CAA ACA TAA AAC AA-3'
	Amplicon size	328 bp

Sequencing was performed on amplicons of bisulfite-treated templates to identify the methylated and unmethylated CpGs with or without 5aza treatment. First, each PCR product was cloned into pGEM-T vector using the pGEM-T Easy Vector System II (Promega) and transformed in JM109 high efficiency competent cells. White colonies containing inserts were selected and cultured overnight, and plasmid DNA was extracted using the QiaPrep Spin Miniprep Kit (Qiagen). Sequencing was done on an ABI 3100 Genetic Analyzer using the ABI PRISM™ BigDye™ Version 1.1 Terminator Cycle Ready Reaction Kit with AmpliTaq DNA Polymerase and an M13R3 primer. Results were downloaded into Sequencher software (Gene Codes, Ann Arbor, MI) to obtain the reverse compliment of each sequence, and both forward and reverse sequences were aligned and analyzed to distinguish unmethylated cytosines from methylated cytosines.

### Western Blot on the AGS cell line

Because of the potential for COX2 inhibitor therapy to overcome COX2 effects, the RNA-based results for COX2 were chosen for follow-up study at the protein-level. Confluent AGS cells with or without EBV were harvested with 0.25% trypsin, washed twice in phosphate-buffered saline, and pelleted by centrifugation. Cells were resuspended in 500 ul NP-40 cell lysis buffer (50 mM Tris-HCl, 150 mM NaCl, 1% NP-40, pH 8.0), incubated on ice for 30 minutes, and spun at 12,000 rpm for 15 minutes at 4°C. Aliquots of lysate (at 50, 100, 150 ug protein per well) were resolved using SDS-PAGE on a tris-glycine 4-20% gradient gel (Invitrogen) and transferred onto a nitrocellulose membrane. COX2 was detected with a 1:5,000 dilution of the monoclonal antibody followed a 1:10,000 dilution of secondary antibody conjugated with alkaline phosphatase (Amersham Biosciences), and visualization with a Typhoon PhosphorImager (Molecular Dynamics). Band density measured semi-quantitatively and normalized to beta actin (ACTB) was compared between infected and uninfected AGS cells.

## Results

### EBV Infection of AGS Gastric Cancer Cells

**Figure 1:**
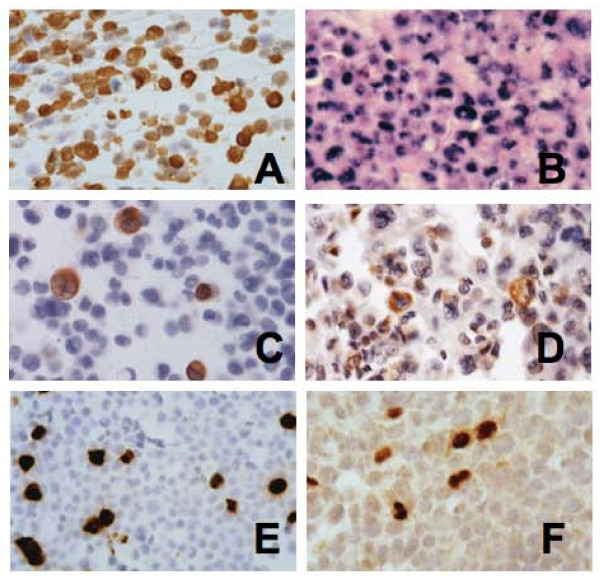
**AGS-B95-HygB cells express latent and lytic viral genes**. A) Immunohistochemical stain for GFP suggests hygromycin B-treated AGS cells were uniformly infected by the engineered B95.8 EBV genome. B) *EBER in situ *hybridization indicates latent infection in >90% of cells. Nuclear *EBER *staining spares the nucleoli, and enlarged nucleoli are a marker of cellular activation. Latent viral proteins LMP1 (C) and LMP2 (D) were expressed focally. Lytic viral proteins, BMRF1 (E) and BZLF1 (F), were expressed in a significant fraction of AGS-B95-HygB cells. (GFP and BMRF1 stains, 800x; LMP1, LMP2, *EBER *and BZLF1 stains, 1200x)

Successful EBV infection of AGS gastric cancer cells was confirmed using six Q-PCR assays targeting disparate segments of the EBV genome. *EBER in situ *hybridization showed no *EBER *expression in the parental "EBV-negative" AGS cells, whereas greater than 90% of AGS-B95-HygB cells were *EBER*-positive and had activated-appearing nuclear morphology (Figure 1). The proliferation rate was increased as shown by confluence of cultured AGS-B95-HygB cells three days prior to parental AGS cells. The infection persisted for at least 4 months as shown by GFP and *EBER *histochemical stains. EBV latent (LMP1 and LMP2A) and lytic (BZLF1 and BMRF1) proteins were not expressed in uninfected AGS cells, whereas ~10% of infected cells expressed LMP1, half the cells expressed LMP2A, and ~35% expressed BMRF1 and BZLF1 proteins implying active viral replication (Figure 1).

### Cellular Gene Expression Differences in EBV-positive versus EBV-negative AGS Cells

**Figure 2:**
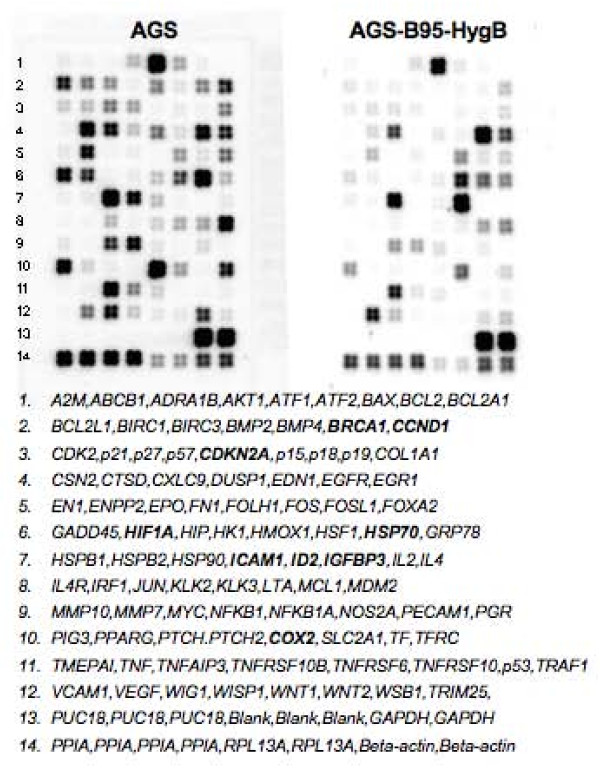
**Gene expression patterns are altered by EBV infection of AGS cells**. Biotin-labeled cDNA probes arrayed in quadruplicate on a nylon membrane hybridize to RNA isolated from AGS and AGS-B95-HygB cells. Chemiluminescent signals indicate dysregulation of selected genes among the 96 on the array as compared with control genes in the last two rows.

Expression levels of 96 cellular genes were analyzed in AGS and AGS-B95-HygB cells using low density microarray analysis with chemiluminescent detection (Figure 2). After normalization to *GAPDH*, pairwise comparison of gene expression levels between the EBV-positive and EBV-negative AGS cells revealed that of the 96 genes on the microarray, 43 were dysregulated at least two-fold after EBV infection. Surprisingly, an EBV-associated increase in expression was observed for only 6 genes (*IGFBP3*, *GADD45, IRF1, Grp78/HSPB1, GLUT1/SLC2A1, TMEPAI*), while decreased expression was observed for the remaining 37 dysregulated genes (*ABCB1,BCL2L1, BIRC2,BMP2, CDKN2A, DUSP1, HIF1A,ICAM1, ID2, NFKB1, COX2, TFRC, VCAM1, WISP1, TRIM25, IL2, BMP4, MDM2, CCND1, CDK2, BAX, p57, p19, CSN2, CXCL9, IL4, JUN, KLK3, LTA, MMP7, PPARG, TNFRSF10B, WIG1, WNT2, PTCH2*).

SYBR Green rtPCR was used to check the microarray findings for 26 of the dysregulated genes and for 12 additional genes in which no significant change was observed on the microarray. Greater than two-fold alteration in mRNA level in infected *versus *uninfected cells was found for 16/26 genes (Table 2). The genes most strongly affected were *IGFBP3 *that was upregulated by 42-fold, and *COX2, BMP4, and ICAM1 *that were downregulated by 35-, 32-, and 22-fold, respectively. The remaining ten microarray-based alterations were not confirmed by rtPCR, suggesting that EBV-related dysregulation of these factors was lower than two-fold. In one instance, there was a major discrepancy: Expression levels of *BCL2L1 *by microarray analysis showed a two-fold *decrease *in infected cells while rtPCR repeatedly showed a five-fold *increase *in *BCL2L1 *mRNA levels in infected cells. Less dramatic discrepancies were found when an additional 12 genes were analyzed by rtPCR, with significant (>2-fold) changes found in the expression of 5/12 genes for which no alteration was observed on the microarray (Table 2).

**Table 2:** Genes Dysregulated in EBV-positive AGS Cells

Gene Name	Gene Symbol	Fold Change*
**Downregulated Genes**		
basic leucine-zipper transcription factor, ATF-like	*BATF*	-38
cyclooxygenase-2	*COX2*	-35
bone morphogenic protein-4	*BMP4*	-32
intercellular adhesion molecule-1	*ICAM1*	-22
trefoil factor-1	*TFF1*	-21
inhibitor of DNA binding-2	*ID2*	-14
heat shock protein-70	*HSP70*	-10
cyclin-dependent kinase-2	*CDK2*	-9
cyclin-D1	*CCND1*	-9
hypoxia inducible factor-1A	*HIF1A*	-9
breast cancer-1	*BRCA1*	-7
nucleostemin	*NU*	-7
cyclin-dependent kinase inhibitor-2A	*CDKN2A/p16*	-6
inhibitor of DNA binding-4	*ID4*	-6
engrailed-1	*EN1*	-5
ATP-binding cassette, sub-family B, member 1	*ABCB1*	-3
MDM2 p53 binding protein	*MDM2*	-3
**Upregulated Genes**		
insulin-like growth factor binding protein-3	*IGFBP3*	+40
tumor necrosis factor receptor-associated factor-1	*TRAF1*	+20
transmembrane prostate androgen-induced protein	*TMEPAI*	+10
tumor necrosis factor, superfamily member-10	*TNFSF10*	+8
B cell lymphoma-2-like-1	*BCL2L1*	+7
baculoviral IAP repeat containing-3	*BIRC3*	+5
hexokinase-1	*HK1*	+5
FOS-like antigen-1	*FOSL1*	+3

*Listed are genes dysregulated by >2-fold using rtPCR

When rtPCR was used to evaluate expression of each of 5 selected genes (*HLTF*, *BATF*, *ID4*, *TFF1*, and *NU) *that were not on the microarray, all except for *HLTF *was downregulated by more than five-fold in the EBV-positive AGS cells (Table 2). *BATF *and *TFF1 *were most significantly affected (38-fold and 21-fold decrease, respectively).

### Demethylation Alters Cellular Gene Expression in AGS and AGS-B95-HygB cells

If gene promoter methylation contributed to EBV-related alterations in gene expression, then demethylation could reverse the effect and perhaps even restore baseline gene expression. To explore this possibility, AGS and AGS-B95-HygB cells were cultured in the presence or absence of 5aza. On the fourth day, RNA was isolated from treated and untreated cells, and rtPCR was performed to measure mRNA levels.

**Figure 3:**
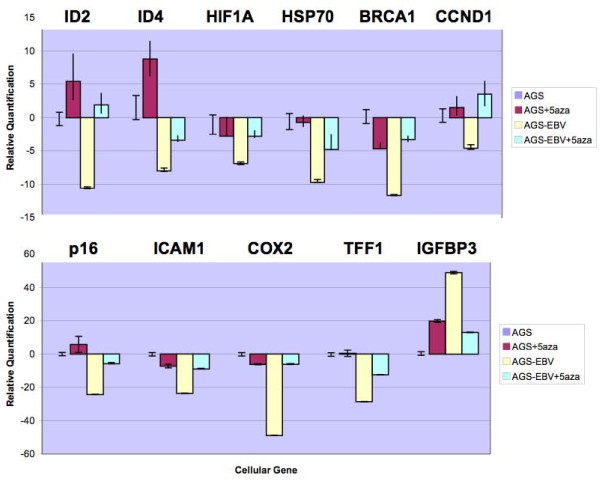
**EBV-related alteration of gene expression is often reversed by treatment with a demethylating agent**. Bar graphs show substantial dysregulation of 11 genes upon EBV infection, as tested by rtPCR, with zero representing baseline expression levels in parental AGS cells. Treatment of uninfected cells with 5-aza-2'-deoxycytidine (5aza) reveals induction of *ID2*, *ID4*, *CCND1*, *CDKN2A (p16) *and *IGFBP3*, suggesting pre-existing promoter methylation. Treatment of infected cells reveals that demethylation can overcome the effect of EBV infection on virus-induced gene dysregulation, at least in part. The most marked drug effect was seen in *ID2, CCND1 *and *COX2 *where demethylation virtually completely reversed the impact of viral infection.

Figure 3 displays the effect of EBV infection on levels of 11 selected genes that encode proteins having a wide range of functions including cell cycle regulation and proliferation (*CDKN2A/p16, CCND1, IGFBP3, HSP70, ID2, ID4)*, DNA repair *(BRCA1, TFF1*), cell adhesion (*ICAM1*), immune response (*COX2*), and angiogenesis (*HIF1A*) [21,23,27,29-37]. Treatment with 5aza had varying effects depending on the gene and infection status. Five of the 11 genes (*ID2, IGFBP3, CDKN2A, ID4*, and *CCND1*) responded to 5aza treatment with *higher *mRNA levels, consistent with promoter methylation in the parental AGS cells. In contrast, *decreased *expression of 4 factors (*BRCA1, HIF1A, ICAM1*, and *COX2*) was observed in response to 5aza treatment, possibly because transcriptional repressors of these genes were demethylated. For the remaining factors (*HSP70 *and *TFF1*), 5aza had no significant effect in AGS cells, suggesting that these genes are not regulated by methylation in the absence of EBV.

In AGS-B95-HygB cells, reduced mRNA levels were observed for 10 genes upon EBV infection, but treatment with 5aza was not sufficient to fully restore expression of any of the genes except for *ID2 *and *CCND1 *where complete reversal of the downregulation was seen (Figure 3). For the remaining 8 genes there was partial restoration of expression upon demethylation.

*IGFBP3 *upregulation in uninfected cells treated with 5aza suggests that an inhibitor of *IGFBP3 *is normally methylated in AGS cells, and that 5aza demethylates the inhibitor to induce *IGFBP3*. EBV infection also strongly induces *IGFBP3*, but subsequent 5aza treatment does not further induce gene expression rather it partly reverses the effect of EBV on *IGFBP3 *induction. Overall, these results suggest that methylation is a mechanism by which EBV influences cellular gene expression, and that demethylation can partially or completely restore baseline expression levels.

### Bisulfite DNA Sequencing Reveals EBV-specific Methylation

Comparisons of methylation patterns were made between 5aza treated and untreated AGS and AGS-B95-HygB cells to explore whether EBV-specific methylation was a plausible cause for dysregulation of five selected genes and to show how patterns of methylation were affected by 5aza. The targeted promoter region for each gene contains multiple CpG dinucleotides: The *CDKN2A *region has 34 sites, *ID4 *has 53 sites, *COX2 *has 25 sites, *ICAM1 *has 35 sites, and *TFF1 *has 14 CpG sites.

**Figure 4:**
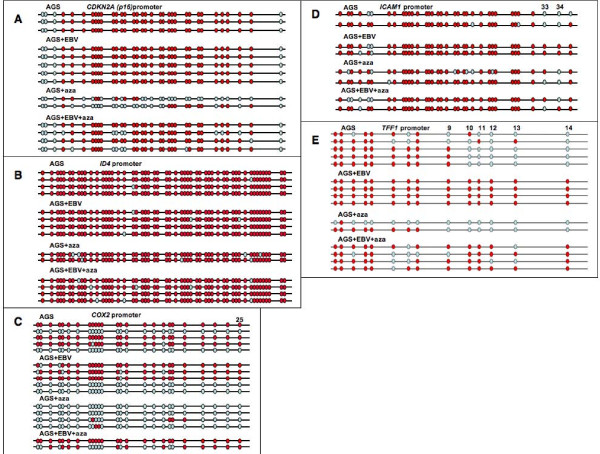
**Promoter methylation is a plausible mechanism of EBV-mediated dysregulation of multiple genes**. Bisulfite sequencing reveals methylation patterns of five genes: A) *CDKN2A (p16)*, B) *ID4*, C) *COX2*, D) *ICAM1*, and E) *TFF1*. Methylation patterns are shown for several clones of each gene with or without EBV infection, and with or without 5-aza-2'-deoxycytidine (aza) treatment. In each clone, methylated sites are dark red ovals whereas unmethylated sites are light blue ovals.

Bisulfite sequencing showed *CDKN2A (p16) *was methylated at 30/34 CpG sites (88%) in both AGS and AGS-B95-HygB cells. After 5aza treatment, only 22-31% of sites remained methylated in AGS cells, whereas 70% of sites remained methylated in AGS-B95-HygB cells (Figure 4A) in concert with restored p16 expression from 24-fold to 6-fold below baseline (Figure 3). The findings imply a dramatic effect of EBV infection on p16 levels, and a more dramatic 5aza-related increase of p16 in infected cells compared to uninfected ones. The dramatic change in expression despite a minimal change in promoter methylation (88 to 70%) after 5aza treatment of infected cells suggests the likelihood of mechanisms other than promoter demethylation.

EBV infection did not increase methylation of *ID4 *since its promoter was already largely methylated before infection occurred. Bisulfite sequence analysis of *ID4 *showed 96-100% methylation in both AGS and AGS-B95-HygB cell lines (Figure 4B). Upon treatment with 5aza, methylation of 92-96% of CpG sites was seen in both infected and uninfected cells, and this seemingly small change in methylation status was accompanied by induction of *ID4 *mRNA. It is feasible that there is a non-methylation mechanism of *ID4 *regulation by EBV.

Interestingly, bisulfite sequencing analysis of *COX2 *showed that individual gene promoters were either completely unmethylated or else largely methylated in AGS and in infected AGS cells, with somewhat different CpG hypermethylation patterns in infected compared to uninfected clones (Figure 4C) accompanied by a marked loss of *COX2 *expression (Figure 3). In the presence of 5aza, there was dramatic demethylation of the *COX2 *promoter in AGS cells with only 0 to 6% methylation. In contrast, the *COX2 *promoter was either completely methylated or only half methylated in various clones of AGS-B95-HygB cells treated with 5aza, suggesting hemi-methylation of alleles in a given cell or else cell-to-cell heterogeneity with respect to *COX2 *methylation patterns. On average, *COX2 *mRNA levels were similar in infected *versus *uninfected cells after treatment with 5aza. (Figure 3). Further work is needed to determine, in any given cell, how methylation status relates to COX2 levels and viral status (e.g. latent or lytic infection). In any case, EBV substantially influences *COX2 *expression and also impacts 5aza effects on its promoter.

*ICAM1 *had distinct methylation patterns in infected *versus *uninfected AGS cells, suggesting that EBV influences expression of this gene via promoter methylation (Figure 4D). Upon EBV infection, methylation of CpG sites increased slightly from 89 to 94%, particularly at sites #33 and 34, and this subtle change was associated with a pronounced decrease in *ICAM1 *expression. Treatment of both cell lines with 5aza yielded demethylation of 6-20% of the CpG sites to achieve similar mRNA levels, which in the infected AGS cells represented a restoration of levels from 24-fold to 9-fold below baseline.

The most dramatic viral-driven changes in promoter methylation were seen in the *TFF1 *gene. The *TFF1 *promoter was 50-64% methylated in AGS cells but 100% methylated in the infected counterpart. In particular, CpG sites #10-14 were methylated upon infection and tended to remain methylated even after attempting to demethylate them with 5aza (Figure 4E.) In EBV-positive AGS cells, 5aza demethylated 14-36% of sites and substantially induced *TFF1 *expression from 30-fold to 12-fold below baseline, whereas in EBV-negative cells demethylation of 43-93% of sites had no effect on *TFF1 *expression. Overall, these findings provide evidence that EBV alters cellular gene expression by direct or indirect effects on promoter methylation, and demethylation can partially overcome viral effects on gene expression.

### Protein-based analysis confirms virus-associated downregulation of COX2 in AGS cells but not in primary gastric adenocarcinoma tissues

**Figure 5:**
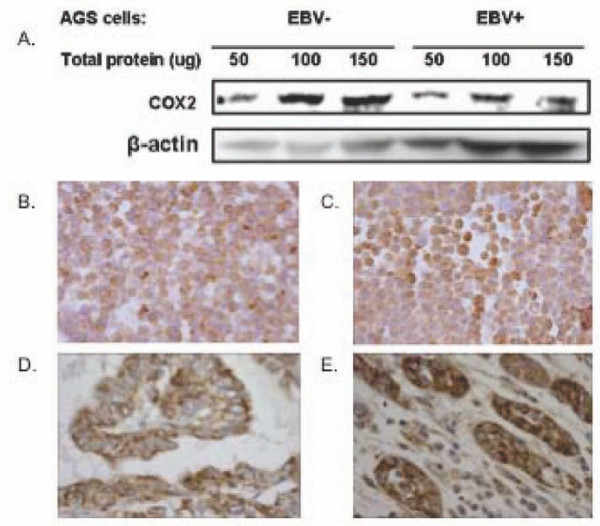
**Western blot but not immunohistochemistry reveals downregulation of COX2 expression in association with EBV infection**. Monclonal antibody was used to semiquantitatively measure COX2 protein. A. Western blot of AGS cell lysates (loaded onto the gel at 50, 100, or 150 ug per lane) showed that EBV positive AGS cells had lower expression of COX2 than did EBV negative cells. However, COX2 immunohistochemistry revealed no discernible difference between EBV negative (B) and EBV positive (C) AGS cell lines. Likewise, COX2 immunohistochemistry of primary gastric adenocarcinoma tissue revealed no visible difference in COX2 expression between EBV negative cancer (D) *versus *EBV positive cancer (E).

COX2 was chosen for analysis at the protein level because of the translational potential for COX2 inhibitor therapy to overcome its effects. Lysates of EBV negative and EBV positive AGS cells were compared in western blot analysis. After normalization, COX2 expression in EBV-positive cells was 1.5 to 2.1 fold (average 1.9) lower than in EBV negative cells, confirming the RNA-based finding that *COX2 *levels are downregulated by EBV in this gastric epithelial cell line. In histologic sections of gastric adenocarcinoma, COX2 protein was localized to the cytoplasm and surface membrane of malignant cells, without significant staining of the nucleus. COX2 was also expressed in a fraction of tumor-infiltrating lymphoid cells. In *EBER*-negative cancers (n = 9), malignant cells had a mean proportion score of 3.3 and intensity score of 1.8, while EBV-positive cancers had a mean proportion score of 3.5 and intensity of 2.4. Total scores were not significantly different in relation to EBV status (p = 0.07). Likewise, immunohistochemistry of AGS cells did not exhibit a visible difference in COX2 expression in relation to EBV status (Figure 5), suggesting that immunohistochemistry is less sensitive to changes in protein level than is western blot.

## Discussion

Our research suggests that DNA methylation is a mechanism by which EBV alters cellular gene expression, and that virus-related methylation can be reversed, at least in part, by a demethylating agent. Since gross chromosomal abnormalities and microsatellite instability are less frequently detected in EBV-associated gastric cancer compared to EBV negative cancer, it is feasible that DNA hypermethylation is a primary driver of virus-related oncogenesis that can be capitalized upon in designing therapeutic interventions [19,21].

It is difficult to analyze the natural effect of EBV infection on gastric cells because laboratory models only partially reflect the effects of EBV infection occurring *in vivo*. For example, LMP1 expression is unusual in naturally infected tumors whereas LMP1 was expressed in 10% of our infected AGS cells [2,63-71]. Low level lytic replication is sometimes reported in naturally infected gastric cancers while it is regularly reported in infected gastric cancer cell lines including our AGS-B95-HygB line [2,65-69,72]. Other limitations of this study are that only one gastric cell line was examined and the demethylating agent was applied for only three days. Nevertheless, our pilot work showing effects of EBV infection on cellular gene expression and promoter methylation in AGS cells encourages further investigation of the prospect of using demethylating agents to overcome virus-associated effects *in vivo*.

The observed viral replication in infected AGS cells might be aided by EBV-mediated downregulation of *CCND1 *and *ID2 *to achieve S-phase-like cellular conditions that are favorable for viral replication [73]. One of the EBV lytic proteins, *BMRF1*, was previously shown to upregulate gastrin [74], and high levels of gastrin are implicated in gastric tumor development [75]. Lytic infection is reportedly induced by 5aza [45], suggesting possible pathways of 5aza effect beyond demethylating promoters. Khan *et al *showed *TFF1 *expression is directly induced by gastrin [76], so 5aza-mediated reversal of *TFF1 *loss might be explained in part by indirect effects on viral replication and gastrin induction in addition to the observed demethylation of the *TFF1 *promoter. *TFF1*, which encodes trefoil factor 1, is suggested to be a tumor suppressor gene that is lost or methylated in many gastric cancers [77]. Interestingly, prior studies suggest that EBV-related hypermethylation is stimulated by pre-existing promoter methylation [19,21,29], and our data on *TFF1 *as well as *ICAM1 *promoters would support this characteristic.

Prior studies showed loss of ID4 expression in about 30% of gastric cancer as well as a means to upregulate ID4 using 5aza in gastric cell lines [32]. Our work shed light on the impact of EBV infection by demonstrating strong downregulation of *ID2 *and *ID4 *upon infection. ID2 and ID4 are members of a family of "inhibitor of differentiation" transcriptional regulators. In breast cancer, methylation-related silencing of *ID4 *is a poor prognostic indicator, and demethylation is proposed as a means of overcoming *ID4 *repression [78]. In our own demethylation studies, we showed that *ID2 *and *ID4 *silencing could be reversed by 5aza in AGS cells.

*COX2 *encodes an enzyme critical for prostaglandin production that mediates inflammation in the gastrointestinal tract. *COX2 *may contribute to carcinogenesis by promoting apoptosis resistance, angiogenesis, invasiveness, and by affecting host immunity [79-88]. *COX2 *gene silencing by hypermethylation in gastric carcinoma cells was previously reported [18,89,90]. Our data show that EBV infection causes a 50-fold decrease in *COX2 *mRNA levels in infected compared to uninfected AGS cells. At the protein level, western blot confirmed downregulation of COX2 in EBV-infected AGS cells although the magnitude of change was less dramatic at about two-fold. Nearly complete restoration of baseline mRNA expression upon demethylation suggests a potential pharmacologic means of reversing the viral effect. The clinical implications of such a finding are intriguing given the reported beneficial effects of COX2 inhibition on incidence, recurrence and outcome of gastrointestinal malignancy [88,91]. Follow-up studies are warranted to explore if COX2 testing of gastric cancer tissue identifies patients most likely to benefit from COX2 inhibition. RNA-based assays may be more informative than immunohistochemistry given our evidence that immunostains were uniquely incapable of quantifying changes in COX2 levels in response to EBV infection of AGS cells. Another group found that COX2 immunostain results were prognostic even though they were not EBV-associated in a series of patients treated for gastric cancer [92].

Heritable polymorphisms in *COX2 *and in *IGFBP3 *have been reported to affect the risk of developing gastric cancer [91,93,94]. *IGFBP3 *is thought to reduce gastric cancer metastasis by sequestering IGFs to prevent them from triggering receptor tyrosine kinases [95]. Methylation of the *IGFBP3 *promoter is found in 67% of gastric cancers, and its silencing is predicted to increase tumor aggressiveness [96]. Our data on upregulation of *IGFBP3 *in response to 5aza treatment confirms a previous study done on uninfected AGS cells [43]. Interestingly, Lee *et al *reported that high *IGFBP3 *expression is a positive predictive marker for response to the antineoplastic drugs paclitaxel and etoposide, suggesting that EBV-infected cancers overexpressing *IGFBP3 *might be particularly susceptible to these chemotherapeutic agents [97]. If confirmed, these insights could impact tumor classification schemes and assist in managing patients with gastric cancer.

## Conclusions

EBV infection had a profound effect on expression of *IGFBP3*. Unlike *IGFBP3 *transcripts which were upregulated, most of the tested gene were *downregulated *by EBV infection. Methylation may be a common mechanism for diverse effects of viral infection. To the extent that viral infection is associated with methylation *in vivo*, demethylating agents could provide a unified therapeutic approach to overcoming viral effects.

Demethylating agents are already used clinically for managing certain neoplasms [98], and their efficacy in EBV-related gastric cancer should be considered. Our pilot data shows frequent and substantial restoration of multiple transcripts in 5aza-treated, infected cells. Pilot sequencing data identify selective effects of virus infection and 5aza treatment on promoter methylation, providing an impetus for further work to characterize viral-mediated effects and to understand how treatments might be devised to overcome viral effects in tumor cells.

## Competing interests

MLG serves on advisory board of Generation Health, and is a consultant for McKesson, Abbott Laboratories, Roche Molecular Diagnostics, and Scientia Advisors.

## Authors' contributions

All authors read and approved the final manuscript. JLR designed experiments, contributed and analyzed data, and drafted the manuscript. RJJ prepared cell lines and revised the manuscript. SCK conceived the idea, provided access to cell lines, and revised the manuscript. AGR designed methylation assays and revised the manuscript. WT validated and applied histochemical stains, and revised the manuscript. ERWK validated and applied COX2 immunostains, and revised the manuscript. WBC participated in study design and revised the manuscript. MLG designed experiments, supervised benchwork, interpreted data, and revised the manuscript.
